# Association of vascular endothelial growth factor polymorphisms with polycystic ovarian syndrome risk: a meta-analysis

**DOI:** 10.1186/s12958-020-00577-0

**Published:** 2020-03-09

**Authors:** Jiahui Zhao, Da Li, Huaiyun Tang, Lisha Tang

**Affiliations:** 1 Department of Reproductive Medicine, Lianyungang Maternal and Child Health Hospital, NO.669 Qindongmen Road, Lianyungang, 222001 Jiangsu Province China; 2 Department of Vascular Surgery, the First People’s Hospital of Lianyungang, NO.182 North Tongguan Road, Lianyungang, Jiangsu Province 222002 China

**Keywords:** VEGF, Polymorphism, Meta-analysis, Polycystic ovarian syndrome

## Abstract

**Background:**

Polycystic ovarian syndrome (PCOS) is a multi-gene hereditary disorder caused by the interaction of certain gene variation with environmental factors. Previous studies have shown that vascular endothelial growth factor (VEGF) gene polymorphisms are associated with the risk of polycystic ovarian syndrome. However, the results of these studies remain controversial. We performed the present meta-analysis aiming to further investigate the potential relationship between VEGF polymorphisms and susceptibility to PCOS.

**Methods:**

The following databases were systematically searched: PubMed, EMBASE, Web of Science (WOS), China National Knowledge Infrastructure (CNKI), and Wanfang Databases. The correlation between VEGF polymorphisms and PCOS risk was assessed by calculating pooled odds ratios (ORs) and their 95% confidence intervals (95% CIs). Subgroup analyses stratified by ethnicity and source of control were also conducted. Besides, trial sequential analysis (TSA) was done to verify the reliability of the pooled results.

**Results:**

10 relevant case-control studies were incorporated in this meta-analysis, involving 1347 PCOS cases and 1378 controls. The VEGF rs2010963 polymorphism was associated with decreased PCOS risk in the whole population and the Asian populations. The VEGF rs3025039 polymorphism was associated with decreased PCOS susceptibility and the Asian populations, but increased risk of PCOS was observed among the Caucasian populations. In addition, the results of trial sequential analysis (TSA) showed the negative correlation between rs2010963 and PCOS risk, obtained by our meta-analysis, was stable and reliable.

**Conclusion:**

Overall, different VEGF gene polymorphisms may exert different effects on PCOS susceptibility. The VEGF rs2010963 polymorphism decreases PCOS susceptibility in both the whole population and the Asian populations, and VEGF rs3025039 polymorphism causes lower PCOS susceptibility in the whole population and the Asian populations but higher in the Caucasian populations.

## Introduction

Polycystic ovarian syndrome (PCOS) is one of the most common endocrine diseases with complex etiology and pathogenesis, with a prevalence of up to 10% in reproductive age women [1, 2]. It is generally characterized by high androgen, long-term persistent anovulation, and ovarian polycystic changes. Typical clinical manifestations include menstrual disorders, infertility, hypertrichosis, acne, obesity, acanthosisnigricans, and polycystic changes in the ovary detected by ultrasound [3]. PCOS is often associated with a variety of metabolic diseases involving patients with varying degrees of insulin resistance, hyperinsulinemia and hyperlipidemia [4]. These metabolic disorders make PCOS patients susceptible to type 2 diabetes, as well as cardiovascular and cerebrovascular diseases [5]. PCOS accounts for approximately 75% of anovulatory infertility [6, 7]. The early abortion rate in PCOS patients is as high as 30 to 50%, and the occurrence of recurrent pregnancy loss is also up to 50%, thus seriously affecting the outcome of pregnancy [8, 9]. The pathogenesis of PCOS, complicated and multifactorial, has not yet been fully elucidated. Currently, it is generally believed that PCOS may be a multi-gene hereditary disorder caused by the interaction of certain genes with environmental factors. Mounting evidence exists that certain genetic polymorphisms play an important role in the pathogenesis of PCOS [10–13].

Vascular endothelial growth factor (VEGF), a homodimeric glycoprotein hormone first purified by Ferrara et al. from the bovine pituitary follicular stellate cells in vitro, is the most active angiogenic factor ever known, which can maintain the differentiation state of vascular endothelial cells and improve microvascular permeability, and play a powerful regulatory role in the occurrence and development of various diseases [14, 15]. It is closely related to the pathogenesis of PCOS, and studies have reported a significant rise in serum level of VEGF in the PCOS patients [16–18]. Additionally, the overexpression of VEGF gene in the polycystic ovarian stroma also reveals the critical role of this gene in the pathophysiology of PCOS [19].

Recent researches have focused on the correlation between VEGF gene polymorphism and PCOS, with rs2010963 (G > C), rs833061 (T > C), rs3025039 (C > T), rs699947 (A > C), and rs1570360 (A > G) extensively investigated for their potential relationship to PCOS risk [20–29]. However, due to different ethnicities and small study sample size, evidence obtained from the different studies remains inconclusive and their results are even controversial. Therefore, we undertook this meta-analysis to explore more comprehensively the relationship between VEGF gene polymorphism and the risk of PCOS by combining existing research results.

## Materials and methods

### Literature search

After formulating the search strategy, we systematically searched the databases of PubMed, EMBASE, Web of Science (WOS), Chinese National Knowledge Infrastructure (CNKI) and Wan Fang to collect potentially eligible case-control studies related to VEGF gene polymorphisms and the risk of PCOS. The last retrieval was conducted on October 1, 2019. The search terms were as follows: (“VEGF” OR “vascular endothelial growth factor”) and (“PCOS” OR “Polycystic ovary syndrome”) and (“mutation” OR “variant” OR “polymorphism” OR “genotype” OR “allele” OR “gene” OR “snp”). To avoid the omission of relevant research, references in the eligible literature after retrieval were also browsed and manually retrieved.

### Inclusion and exclusion criteria

The studies included in this meta-analysis must meet the following criteria: (a) Case-control studies covered the relationship between VEGF gene polymorphisms and risk of PCOS. (b) The sample size, genotype distribution, and genotype frequency of the case and control groups were directly provided in the document, or the literature contained available original data that could be converted into OR value and 95%CI for the case and control groups. (c) The diagnosis of PCOS patients in the case group must conform to one of the following three diagnostic criteria: NIH, Rotterdam, and AE-PCOS Society [30–32] (d). The inclusion criteria for the control group were clear and definite. (e) If multiple articles based on the same case and control population were published by the same author or research center, only relevant researches in the latest publication time would be incorporated.

Studies were excluded on the basis of the following criteria: (a) There was no clear definition of polycystic ovary syndrome in the literature, and no clear diagnostic criteria were available for enrolled patients in the case group. (b) The specific genotype data of the case group and the control group were not provided, or the genotype frequency of the case group and the control group could not be calculated by the original data in the literature. (c) Conference papers, case reports, literature reviews or animal researches were ruled out.

### Data extraction

Two reviewers (Jiahui Zhao and Da Li) read screened independently the eligible relevant literature according to the inclusion and exclusion criteria. When the two researchers disagreed about whether or not a study should be included, they would try to resolve it through discussion. If they could not reach a consensus, the final decision would be made by a third researcher (Lisha Tang). For the included literature after screening, the following data were mainly extracted: first author, publication year, nationality, ethnicity, PCOS diagnostic criteria adopted, inclusion criteria of the control group, source of control, genotyping method, single nucleotide polymorphisms (SNPs) investigated in the study, sample size and genotype distribution of the case group and the control group.

### Quality assessment

The literature quality was evaluated by Newcastle-Ottawa Scale (NOS) [33]. The NOS evaluation criterion of case-control study included 9 items (1 point for each item, 9 points in total). The specific scores involved: (1) selection of the case group and control group (4 points) based on appropriate definition and diagnosis of the case, representativeness of the case, selection of control, and definition of control; (2) comparability between the case group and control group (2 points); and (3) exposure evaluation of the case group and control group (4 points). If the study satisfied the evaluation score at 7 points or above, the quality of the literature was thought to be high; if the research evaluation score ranged 4–6, the quality of the literature was medium; and if the score wass < 4, the literature quality was considered low.

### Statistical analysis

Meta-analysis was mainly performed using Revman5.3 software by the Cochrane Collaboration. The strength of association between VEGF polymorphisms and susceptibility of PCOS was evaluated by calculating Odds ratios (OR) and its 95% confidence intervals (CIs) under five gene comparison models. For example, the correlation between rs2010963 (G > C) polymorphism and PCOS risk was measured and assessed under the following models: allele model (C vs. G), dominant model (GC + CC vs. GG), heterozygous model (GC vs. GG), recessive model (CC vs. GC + GG) and homozygous model (CC vs. GG). The heterogeneity between studies was quantitatively determined by I^2^ statistic. If I^2^ was lower than 50%, which indicated that the heterogeneity was low, the fixed-effect model would be applied for meta-analysis. If statistical heterogeneity between studies existed (I^2^ > 50%), the source of heterogeneity would be further analyzed, and the random effect model would be adopted. In addition to overall analysis, subgroup analyses stratified by ethnicity and source of control were also performed. Since all of the included literature adopted the Rotterdam diagnostic criteria for the enrolled patients of case groups, there was no subgroup analyses based on different diagnostic criteria. If there was only one article in a subgroup, no separate analysis would be performed.

The possible publication bias was first evaluated by observing the symmetry of the funnel plots. The Stata14.0 software (STATA Corporation, College Station, TX) was further applied to perform the Begg’s and Egger’s test procedures to assess potential publication bias. The genotype distribution of the control group was calculated by χ^2^ test to ascertain whether the control group was consistent with Hardy-Weinberg equilibrium (HWE).

Traditional meta-analysis methods have no way to assess the adequacy of the overall sample size of the included studies. When the overall sample size is not sufficient, the final pooled results obtained are not reliable enough, especially the positive results obtained by meta-analysis. Statistical results based on insufficient sample size increase the likelihood of false positive results (Type 1 error). Therefore, in the present study, we adopted trial sequential analysis (TSA) method to determine whether the overall sample size of the included studies was sufficient and whether the overall results obtained were reliable. Trial sequential analysis (TSA) was performed by using the TSA v0.9.5.10 Beta software [34]. For the positive results obtained from meta-analysis, TSA method was applied to analyze the reliability of the results and examine whether the sample size was sufficient. If the cumulative Z curve crossed trial sequential monitoring boundary, the result was considered stable and reliable. The sufficiency of overall sample size was estimated based on whether the cumulative Z-curve exceeded the required information size (RIS).

### Ethical approval

The meta-analysis was performed by summarizing data from previous studies. Therefore, no ethical approval was required.

## Results

### Study selection and characteristics

A total of 129 articles were obtained through initial literature retrieval. After excluding 25 duplicates, the remaining 104 papers were filtrated by reading their titles and abstracts. After further screening, totally 12 papers entered the full-text acquisition and detailed screening. After excluding a repetitive study based on the same case group and another article focusing on the association between VEGF gene polymorphisms and insulin resistance in patients with PCOS, 10 articles met the inclusion criteria and eventually merged into the study. A total of 1347 PCOS cases and 1378 controls were included in the 10 included studies [20–29]. This analysis spanned seven regions and two ethnic groups, Caucasian and Asian. In all the incorporated studies, the diagnostic criteria of PCOS patients in the case group were Rotterdam criteria, and the inclusion criteria of the control group were clearly explained. The NOS score of all the included studies was higher than 6 points, and most of them explored the relationship between multiple SNPs and PCOS susceptibility. Therefore, in the final pooled analysis, 6 studies were eligible for VEGF gene rs2010963 polymorphism and PCOS risk (952 cases and 960 controls), 6 for rs833061 polymorphism and PCOS risk (936 cases and 1010 controls), 5 for rs3025039 polymorphism and PCOS risk (769 cases and 779 controls), 5 for rs699947 polymorphism and PCOS risk (779 cases and 834controls), and 5 for rs1570360 polymorphism and PCOS risk (752 cases and 779 controls).

**Fig. 1:**
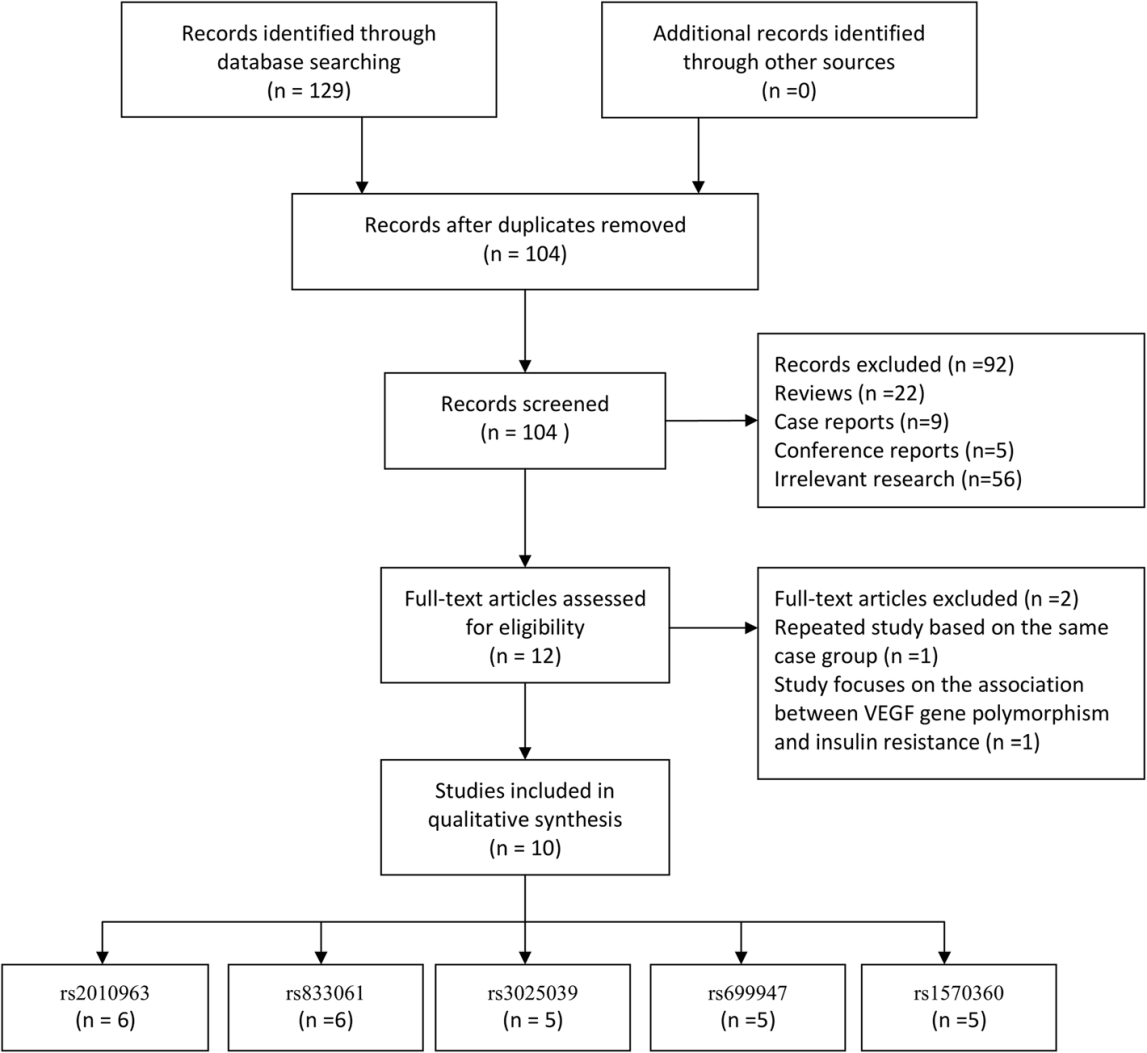
Flow diagram of selection and inclusion process

The flowchart of selection and inclusion process is displayed in Fig. 1, and the general characteristics of all included studies in Table 1. Table 2 shows the detailed genotype distributions of each group and the *p* value of Hardy-Weinberg equilibrium (HWE) in each control group. The Newcastle-Ottawa Scale scores for all the included studies are shown in Table 3.

**Table 1:** Characteristics of included studies

Author	Year	Region	Ethnicity	PCOS Diagnostic criteria	Inclusion criteria for the control group	Control source	Genotyping method	SNP	NOS
Almawi [20]	2016	Bahrain	Caucasian	Rotterdam	age-and ethnically-matched; testosterone levels were within range (0.4–3.5 nmol/L) in the menstrual cycle follicular phase; no metabolic or endocrine diseases; BMI were between 18 and 45 kg/m^2^;without recent illness; without treatment may affect carbohydrate metabolism or hormonal levels within 3 months; without using anti-hypertensive, oral contraceptive, anti-inflammatory and lipid-lowering drugs	PB	RT-PCR	rs2010963 rs833061 rs3025039 rs699947 rs1570360	8
Bao [21]	2019	India	Asian	Rotterdam	age-and ethnically-matched; testosterone levels were within range (0.4–3.5 nmol/L) in the menstrual cycle follicular phase; no metabolic or endocrine diseases; BMI were between 18 and 45 kg/m^2^;without recent illness; without using anti-hypertensive, oral contraceptive, anti-inflammatory and lipid-lowering drugs	PB	RT-PCR	rs2010963 rs833061 rs3025039 rs699947 rs1570360	6
Ben Salem [22]	2016	Tunisia	Caucasian	Rotterdam	women with regular menstrual cycles and no evidence of hirsutism, acne, alopecia, or endocrinopathies; without treatment may affect carbohydrate metabolism or hormonal levels within 3 months	PB	RT-PCR	rs833061 rs3025039 rs699947 rs1570360	7
Ding [28]	2009	China	Asian	Rotterdam	women with regular menstrual cycles; with normal ovarian morphology; without treatment may affect carbohydrate metabolism or hormonal levels within 3 months; no metabolic or endocrine diseases;	HB	PCR	rs3025039	6
Gomes [23]	2019	Brazil	Caucasian	Rotterdam	age- matched; fertile women with no history of hyperandrogenism, menstrual dysfunction, infertility, or sonographic signal of PCOS; without using contraceptives, antiandrogens, statins, glucocorticoids or infertility medications within 6 months	PB	PCR-RFLP (rs699947) RT-PCR (rs1570360)	rs699947 rs1570360	6
Guruvaiah [24]	2014	India	Asian	Rotterdam	fertile women with regular menstrual cycles and had a successful pregnancy record; with normal ovarian morphology confirmed by ultrasound	PB	PCR	rs2010963 rs833061	7
Huang [25]	2019	China	Asian	Rotterdam	women with normal menstrual cycles and had at least one successful pregnancy history; without endocrine disorders, family history of diabetes or sonographic signs of PCOS	HB	PCR-LDR	rs2010963 rs833061	7
Lee [26]	2008	Korea	Asian	Rotterdam	BMI-matched; with regular menstrual cycles; with normal ovarian morphology confirmed by ultrasound	PB	PCR	rs2010963 rs3025039	6
Li [29]	2014	China	Asian	Rotterdam	fertile women with regular menstrual cycles	HB	PCR-RFLP	rs1570360	6
Vural [27]	2009	Turkey	Caucasian	Rotterdam	fertile women without hyperandrogenism, history of menstrual dysfunction, infertility or sonographic signs of PCOS.	PB	PCR	rs2010963 rs833061 rs699947	6

*PCR* polymerase chain reaction, *RFLP* restriction fragment length polymorphism, *RT-PCR* real time polymerase chain reaction, *PCR-LDR* polymerase chain reaction-ligase detection reaction, *NOS* Newcastle–Ottawa scale, *PB* Population-based, *HB* Hospital-based, *BMI* body mass index, *PCOS* polycystic ovary syndrome

**Table 2:** Genotype distribution and HWE

SNP	Author	Sample size		Case Genotype			Control genotype			HWE
		Case	Control	GG	GC	CC	GG	GC	CC	Y/N(***p***)
**rs2010963**	Almawi	382	393	183	142	57	161	190	42	0.45
	Bao	55	52	28	11	16	26	12	14	0.47
	Guruvaiah	126	130	70	46	10	52	59	19	0.73
	Huang	118	130	60	45	13	47	64	19	0.71
	Lee	134	100	46	60	26	34	45	20	0.47
	Vural	137	155	90	44	3	112	39	4	0.78
**SNP**	**Author**	**Sample size**		**Case Genotype**			**Control genotype**			**HWE**
		**Case**	**Control**	**TT**	**TC**	**CC**	**TT**	**TC**	**CC**	**Y/N(*p*)**
**rs833061**	Almawi	382	393	130	174	78	132	190	71	0.91
	Bao	55	52	32	9	14	24	11	17	0.06
	Ben Salem	118	150	33	55	30	42	76	32	0.82
	Guruvaiah	126	130	40	59	27	33	72	25	0.20
	Huang	118	130	63	45	10	80	42	8	0.44
	Vural	137	155	55	64	18	52	74	29	0.77
**SNP**	**Author**	**Sample size**		**Case Genotype**			**Control genotype**			**HWE**
		**Case**	**Control**	**CC**	**CT**	**TT**	**CC**	**CT**	**TT**	**Y/N(*p*)**
**rs3025039**	Almawi	382	393	296	81	5	318	68	7	0.78
	Bao	55	52	31	9	15	24	11	17	0.10
	Ben Salem	118	150	89	27	2	127	19	4	0.005
	Ding	80	84	45	35	0	54	30	0	0.04
	Lee	134	100	89	35	4	59	31	10	0.06
**SNP**	**Author**	**Sample size**		**Case Genotype**			**Control genotype**			**HWE**
		**Case**	**Control**	**AA**	**AC**	**CC**	**AA**	**AC**	**CC**	**Y/N(*p*)**
**rs699947**	Almawi	382	393	135	183	64	165	178	50	0.48
	Bao	55	52	34	5	16	26	09	17	0.17
	Ben Salem	118	150	35	63	20	45	77	28	0.62
	Gomes	87	84	27	38	22	18	41	25	0.70
	Vural	137	155	52	63	22	52	78	25	0.64
**SNP**	**Author**	**Sample size**		**Case Genotype**			**Control genotype**			**HWE**
		**Case**	**Control**	**AA**	**AG**	**GG**	**AA**	**AG**	**GG**	**Y/N(*p*)**
**rs1570360**	Almawi	382	393	197	140	45	218	131	44	0.09
	Bao	55	52	24	13	18	30	9	13	0.03
	Ben Salem	118	150	57	42	19	75	57	18	0.17
	Gomes	87	84	56	24	7	52	31	1	0.12
	Li	110	100	78	29	3	65	30	5	0.53

*HWE* Hardy–Weinberg equilibrium

**Table 3:** Quality assessment using the Newcastle–Ottawa scale

Study	Adequacy of Case Definition	Representativeness of the Cases	Selection of Controls	Definition of Controls	Compara- bility	Ascertainment of exposure	Same method of ascertainment	Non-Response rate	score
Almawi 2016 [20]	*	*	*	*	*	*	*	*	8
Bao 2019 [21]	*		*	*		*	*	*	6
Ben Salem 2016 [22]	*	*	*	*		*	*	*	7
Ding 2009 [28]	*		*	*		*	*	*	6
Gomes 2019 [23]	*		*	*		*	*	*	6
Guruvaiah 2014 [24]	*	*	*	*		*	*	*	7
Huang 2019 [25]	*	*	*	*		*	*	*	7
Lee 2008 [26]	*	*		*		*	*	*	6
Li 2014 [29]	*	*		*		*	*	*	6
Vural 2009 [27]	*	*		*		*	*	*	6

### Meta-analysis results

#### Meta-analysis of association between VEGF gene rs2010963 polymorphism and PCOS susceptibility

**Fig. 2:**
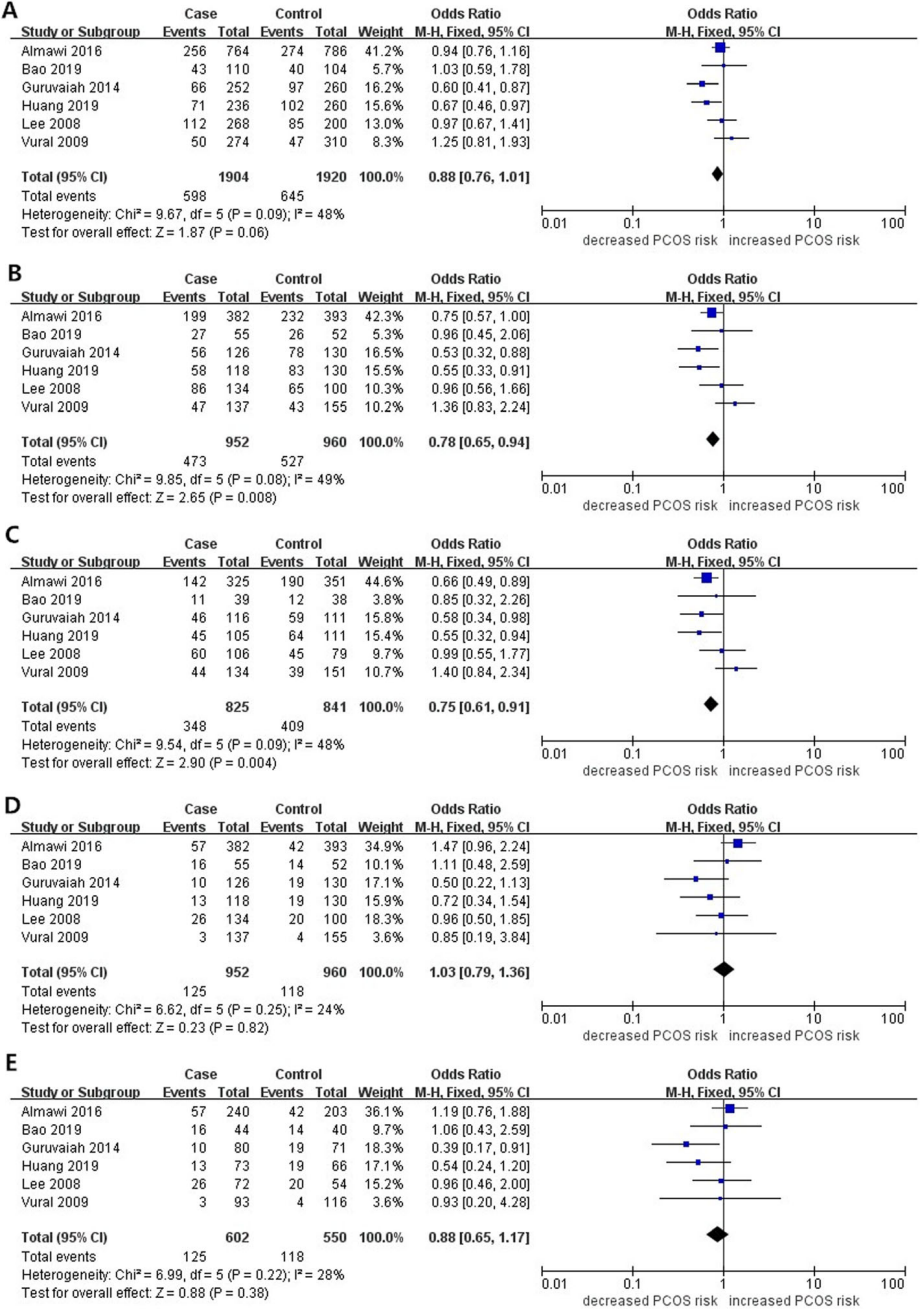
Forest plots for association between VEGF gene rs2010963 polymorphism and PCOS susceptibility in different genetic models (**a**: allele model **b**:dominant model **c**:heterozygous model **d**:recessive model **e**:homozygous model)

The main results of the overall and subgroup meta-analysis for VEGF gene polymorphisms and PCOS susceptibility are presented in Table 4. Six studies had 952 cases and 960 controls for VEGF rs2010963 polymorphism. Four of them were based on the Asian populations and two on the Caucasian populations. Overall, the pooled results based on the whole population revealed significant correlation between VEGF rs2010963 variation and decreased PCOS susceptibility under 2 gene comparison models (Dominant model: OR = 0.78, 95%CI: 0.65–0.94, *P* = 0.008, I^2^ = 49%; Heterozygote model: OR = 0.75, 95%CI: 0.61–0.91, *P* = 0.04, I^2^ = 48%). When the studies were stratified by ethnicity, significantly decreased risk of PCOS was observed in the Asian populations under 3 gene comparison models (Allele model: OR = 0.76, 95%CI: 0.63–0.93, P = 0.008, I^2^ = 39%; Dominant model: OR = 0.69, 95%CI: 0.50–0.95, *P* = 0.02, I^2^ = 26%; Heterozygote model: OR = 0.68, 95%CI: 0.50–0.92, *P* = 0.01, I^2^ = 0%). No significant association was found between VEGF rs2010963 polymorphism and PCOS susceptibility in the Caucasian ethnicities and stratified subgroup analyses based on source of control. Figure 2 shows the forest plot of the association between VEGF gene rs2010963 polymorphism and PCOS susceptibility.

**Table 4:** Main results of overall and subgroup analyses for VEGF polymorphisms and PCOS

Group/ Subgroup	Sample size (Case/Control)	Allele model			Dominant model			Heterozygote model			Recessive model			Homozygote model		
		OR (95%CI)	***p***	I^**2**^	OR (95%CI)	***p***	I^**2**^	OR (95%CI)	***p***	I^**2**^	OR (95%CI)	***p***	I^**2**^	OR (95%CI)	***p***	I^**2**^
**rs2010963**																
Total	952/960	0.88 [0.76,1.01]	0.06	48%	0.78 [0.65, 0.94]	**0.008**	49%	0.75 [0.61, 0.91]	**0.004**	48%	1.03 [0.79, 1.36]	0.82	24%	0.88 [0.65, 1.17]	0.38	28%
Caucasian	519/548	0.99 [0.82, 1.20]	0.94	24%	0.98 [0.55, 1.73]	0.93	75%	0.93 [0.44, 1.96]	0.85	84%	1.41 [0.93, 2.12]	0.10	0%	1.17 [0.76, 1.80]	0.48	0%
Asian	433/412	0.76 [0.63, 0.93]	**0.008**	39%	0.69 [0.50, 0.95]	**0.02**	26%	0.68 [0.50, 0.92]	**0.01**	0%	0.80 [0.55, 1.16]	0.24	0%	0.68 [0.46, 1.02]	0.06	21%
PB	834/830	0.92 [0.79, 1.06]	0.24	45%	0.82 [0.68, 1.00]	0.29	48%	0.83 [0.59, 1.16]	0.27	51%	1.09 [0.81, 1.47]	0.56	29%	0.95 [0.69, 1.30]	0.73	24%
**rs833061**																
Total	936/1010	0.98 [0.86, 1.12]	0.80	25%	0.93 [0.77,1.12]	0.45	6%	0.91 [0.74,1.11]	0.35	0%	1.06 [0.84,1.33]	0.63	0%	0.97 [0.75,1.26]	0.83	0%
Caucasian	637/698	0.99 [0.84, 1.15]	0.85	22%	0.93 [0.74,1.16]	0.51	0%	0.90 [0.71,1.15]	0.4	0%	1.06 [0.81,1.40]	0.66	27%	1.00 [0.73,1.35]	0.98	28%
Asian	299/312	0.96 [0.68, 1.37]	0.83	51%	0.90 [0.54,1.49]	0.67	54%	0.93 [0.64,1.33]	0.68	47%	1.05 [0.68,1.62]	0.84	0%	0.92 [0.57,1.48]	0.73	0%
PB	818/880	0.95 [0.83, 1.09]	0.48	10%	0.87 [0.71,1.07]	0.18	0%	0.85 [0.68,1.06]	0.14	0%	1.04 [0.82,1.32]	0.74	0%	0.94 [0.72,1.23]	0.63	0%
**rs3025039**																
Total	779/834	0.97 [0.68, 1.39]	0.88	65%	1.07 [0.73, 1.57]	0.73	58%	1.21 [0.95, 1.55]	0.12	42%	0.59 [0.34, 1.02]	0.06	0%	0.56 [0.32, 0.99]	**0.05**	0%
Caucasian	500/543	1.22 [0.93, 1.60]	0.16	0%	1.35 [1.00, 1.83]	**0.05**	10%	1.43 [1.04, 1.95]	**0.03**	33%	0.70 [0.27, 1.82]	0.46	0%	0.76 [0.29, 1.97]	0.57	0%
Asian	269/236	0.79 [0.48, 1.29]	0.35	63%	0.83 [0.49, 1.41]	0.49	52%	0.94 [0.63, 1.39]	0.75	26%	0.54 [0.28, 1.05]	0.07	48%	0.48 [0.24, 0.97]	**0.04**	36%
PB	689/695	0.91 [0.60, 1.39]	0.68	71%	1.00 [0.63, 1.60]	0.99	66%	1.13 [0.72, 1.76]	0.60	55%	0.59 [0.34, 1.02]	0.06	0%	0.56 [0.32, 0.99]	**0.05**	0%
HWE (yes)	571/545	0.79 [0.50,1.25]	0.32	70%	0.84 [0.51,1.40]	0.51	63%	1.06 [0.79,1.41]	0.71	41%	0.58 [0.33,1.04]	0.07	4%	0.55 [0.30,0.99]	**0.05**	0%
**rs699947**																
Total	779/834	1.03 [0.90, 1.19]	0.65	49%	1.03 [0.84, 1.26]	0.78	48%	1.01 [0.81, 1.26]	0.91	37%	1.07 [0.82, 1.38]	0.62	0%	1.06 [0.80, 1.41]	0.69	36%
Caucasian	724/782	1.00 [0.80, 1.25]	0.99	51%	1.07 [0.87, 1.32]	0.53	49%	1.05 [0.83, 1.31]	0.70	30%	1.09 [0.83, 1.43]	0.52	0%	1.11 [0.82, 1.51]	0.49	44%
**rs1570360**																
Total	752/779	1.11 [0.95, 1.30]	0.20	0%	1.15 [0.94, 1.41]	0.17	11%	0.69 [0.37, 1.27]	0.23	82%	1.23 [0.89, 1.70]	0.21	15%	1.29 [0.92, 1.80]	0.14	13%
Caucasian	587/627	1.12 [0.94, 1.34]	0.19	0%	1.18 [0.94, 1.49]	0.14	0%	0.58 [0.24, 1.37]	0.21	88%	1.26 [0.88, 1.81]	0.21	40%	1.31 [0.90, 1.90]	0.16	23%
Asian	165/152	1.08 [0.53, 2.23]	0.83	73%	1.11 [0.49, 2.53]	0.79	66%	0.95 [0.24, 3.81]	0.95	82%	1.12 [0.55, 2.30]	0.75	27%	1.22 [0.58, 2.57]	0.60	50%
PB	642/679	1.16 [0.98, 1.37]	0.08	0%	1.22 [0.98, 1.52]	0.07	0%	0.75 [0.35, 1.58]	0.44	84%	1.29 [0.92, 1.79]	0.14	13%	1.36 [0.96, 1.93]	0.08	0%
HWE (yes)	697/727	1.08 [0.91,1.27]	0.39	0%	1.12 [0.90,1.38]	0.30	8%	0.56 [0.29,1.09]	0.09	84%	1.19 [0.84,1.69]	0.32	33%	1.23 [0.85,1.76]	0.27	25%

*VEGF* vascular endothelial growth factor, *PCOS* polycystic ovary syndrome, *OR* odds ratio, *CI* confidence interval, *NA* not available, *HB* hospital-based, *PB* population-based. The bold values represent statistically significant differences

#### Meta-analysis of association between VEGF gene rs833061 polymorphism and PCOS susceptibility

Six studies had 936 cases and 1010 controls for VEGF rs2010963 polymorphism, 3 of which were based on the Asian populations and another 3 on the Caucasian populations. There was no significant correlation found between VEGF gene rs833061 polymorphism and risk of PCOS, whether based on aggregate analysis of all populations or subgroup analyses based on different ethnicities and sources of control.

#### Meta-analysis of association between VEGF gene rs3025039 polymorphism and PCOS susceptibility

Five studies had 769 cases and 779 controls for VEGF rs3025039 polymorphism, 3 of which were based on the Asian populations and 2 on the Caucasian populations. Overall, the pooled results based on the whole population revealed significantly negative correlation between VEGF rs2010963 polymorphism and PCOS risk under Homozygote model (OR = 0.56, 95%CI: 0.32–0.99, *P* = 0.05, I^2^ = 0%), Based on ethnic stratification, significantly increased risk of PCOS was observed in the Caucasian populations under 2 gene comparison models (Dominant model: OR = 1.35, 95%CI: 1.00–0.95, P = 0.05, I^2^ = 10%; Heterozygote model: OR = 1.43, 95%CI: 1.04–1.95, *P* = 0.03, I^2^ = 33%). However, in the Asian populations, there was negative correlation between VEGF rs2010963 polymorphism and PCOS risk under Homozygote model (OR = 0.48, 95%CI: 0.24–0.97, *P* = 0.04, I^2^ = 36%). Moreover, subgroup analysis based on source of control indicated that VEGF rs3025039 polymorphism was related to decreased PCOS risk in the population-based group under Homozygote model (OR = 0.56, 95%CI: 0.32–0.99, *P* = 0.05, I^2^ = 0%).

#### Meta-analysis of association between VEGF gene rs699947 polymorphism and PCOS susceptibility

Five studies had 779 cases and 834 controls for VEGF rs699947 polymorphism. Four of them were based on the Caucasian populations. There was no significant correlation between VEGF gene rs699947 polymorphism and risk of PCOS, whether based on analysis by synthesis of all populations or subgroup analyses based on the Caucasian ethnicities.

#### Meta-analysis of association between VEGF gene rs1570360polymorphism and PCOS susceptibility

Five studies had 752 cases and 779 controls for VEGF rs1570360 polymorphism. Three of them were based on the Caucasian populations and two on the Asian populations. No significant association was identified between VEGF gene rs1570360 polymorphism and risk of PCOS, whether based on aggregate analysis of all populations or subgroup analyses based on different ethnicities and sources of control.

### Publication bias

**Fig. 3:**
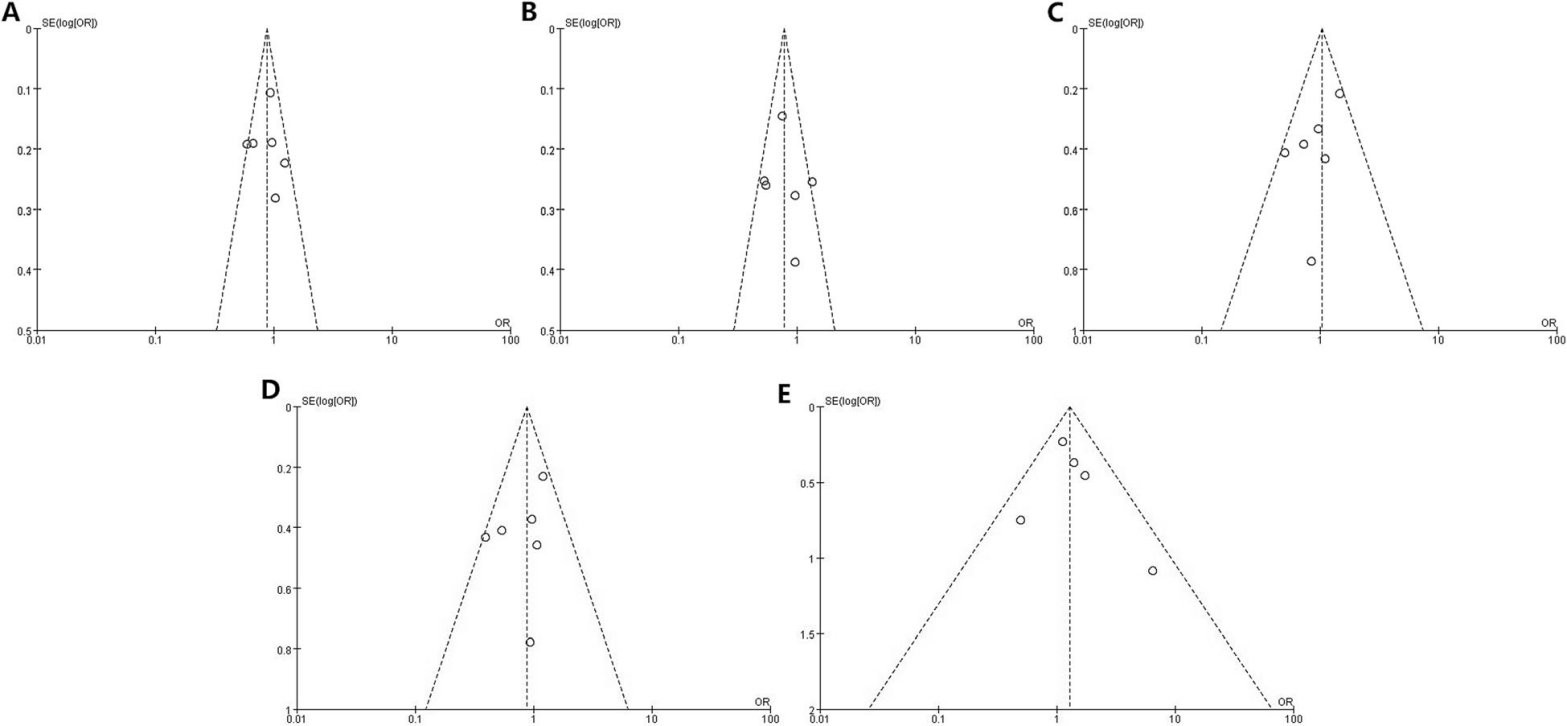
Funnel plot for the association betweenVEGF gene rs2010963 polymorphism and PCOS risk (**a**: allele model **b**:dominant model **c**:heterozygous model **d**: recessive model **e**:homozygous model)

Statistical association was found between VEGF rs2010963 and rs3025039 polymorphisms and PCOS susceptibility. The potential publication bias was first evaluated by observing the funnel plot obtained through Revman5.3 software. Funnel plots showed no conspicuous visual asymmetry (Fig. 3). Begg’s test and Egger’s test were applied to further detect potential publication bias. The results of Begg’s test and Egger’s test also revealed no significant evidence of publication bias. Table 5 summarizes the main results of Begg’s test and Egger’s test.

**Table 5:** Summary of publication bias tests

SNP	Comparison model type	begg’s text		egger’s text	
		z	***p*** value	t	***p*** value
**rs2010963**	Allele model	−0.19	0.851	0.04	0.969
	Dominant model	0.56	0.573	0.48	0.655
	Heterozygote model	0.56	0.576	1.31	0.261
	Recessive model	−0.56	0.573	−2.01	0.115
	Homozygote model	−0.94	0.348	−1.31	0.260
**rs833061**	Allele model	0.19	0.851	−0.50	0.641
	Dominant model	−0.94	0.348	3.15	0.035
	Heterozygote model	−1.32	0.188	−0.45	0.679
	Recessive model	−0.19	0.851	−0.55	0.608
	Homozygote model	−0.19	0.851	−0.33	0.760
**rs3025039**	Allele model	0.49	0.624	−0.01	0.993
	Dominant model	0.00	1.000	−0.22	0.837
	Heterozygote model	0.49	0.624	−0.23	0.835
	Recessive model	−1.36	0.174	−0.66	0.576
	Homozygote model	−0.68	0.497	1.17	0.362
**rs699947**	Allele model	−1.47	0.142	−5.82	0.010
	Dominant model	−1.47	0.142	−5.19	0.014
	Heterozygote model	−0.98	0.327	−3.59	0.037
	Recessive model	−1.47	0.142	−5.07	0.015
	Homozygote model	−1.47	0.142	−10.90	0.002
**rs1570360**	Allele model	0.98	0.327	0.24	0.828
	Dominant model	0.00	1.000	−0.43	0.696
	Heterozygote model	−0.49	0.624	−0.50	0.651
	Recessive model	1.47	0.142	1.49	0.233
	Homozygote model	1.47	0.142	1.43	0.249

### Sensitivity analysis

**Fig. 4:**
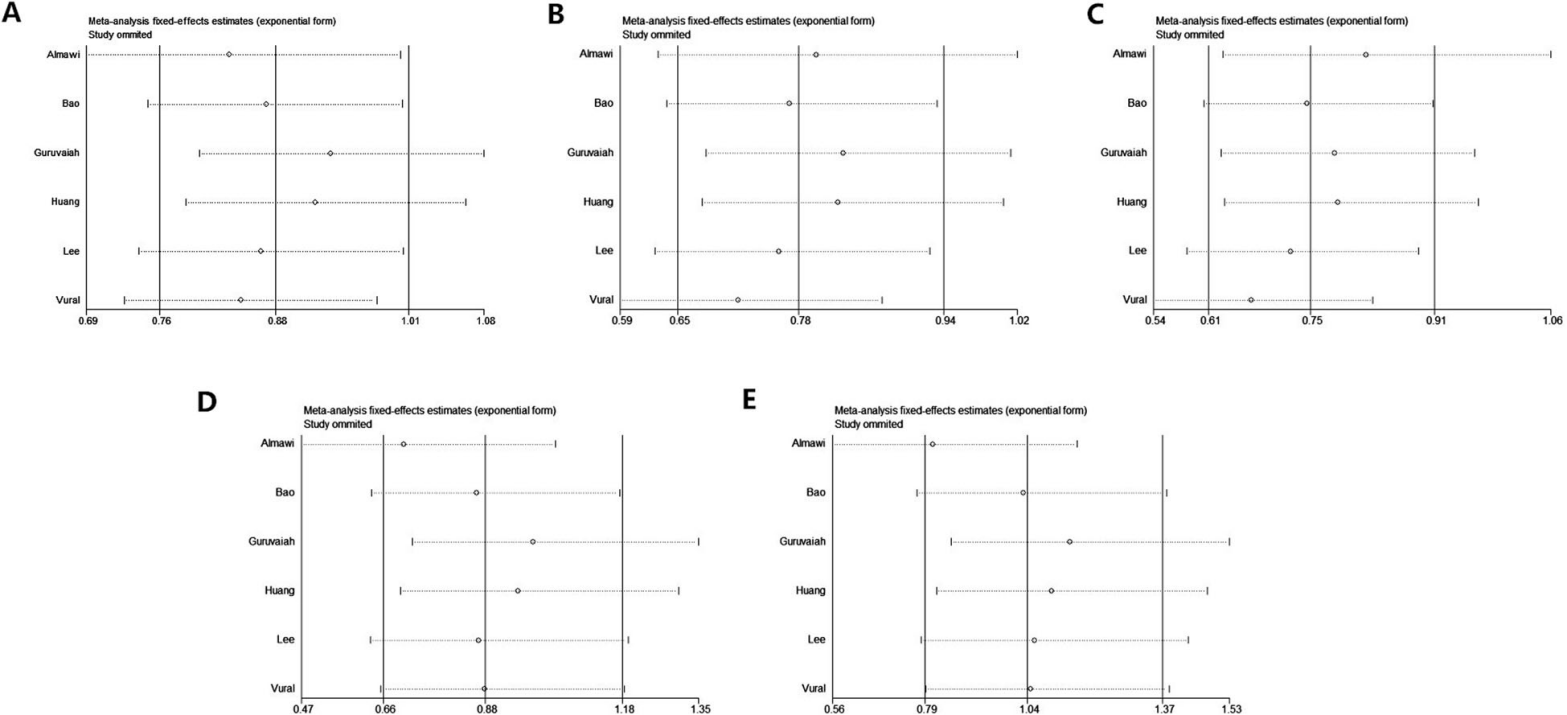
Sensitivity analysis results between VEGF gene rs2010963 polymorphism and PCOS risk (**a**: allele model **b**:dominant model **c**:heterozygous model **d**: recessive model **e**:homozygous model)

Sensitivity analysis was performed to assess the influence of each individual study on the overall pooled results. By sequentially removing individual study, no statistical variation of pooled OR was observed. The results of sensitivity analysis indicated that the pooled ORs of this meta-analysis were stable (Fig. 4).

### Trial sequential analysis

**Fig. 5:**
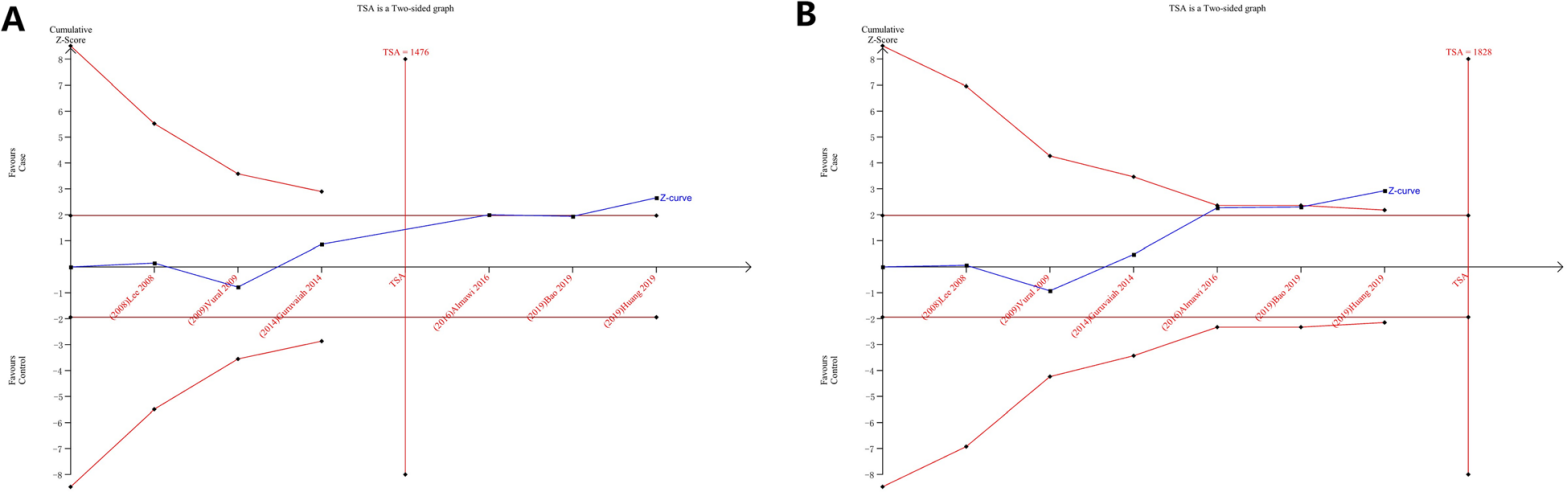
Trial sequential analysis results of the association between VEGF gene rs2010963 polymorphism and PCOS risk (**a**:dominant model **b**: heterozygous model)

Trial sequential analysis was performed for each gene comparison model with statistically significant differences obtained through previous calculation to investigate whether the pooled results we obtained were stable and reliable. Under the dominant model of rs2010963, although the cumulative Z curve did not intersect with the trial sequential monitoring boundary, the cumulative case samples exceeded the required information size (RIS), suggesting that the accumulated sample size was sufficient and the pooled positive result was credible. Under the heterozygote model of rs2010963, the cumulative Z curve passed through the traditional boundary threshold, meanwhile crossed the trial sequential monitoring boundary, and confirmed a reliable conclusion although the cumulative sample size did not transcend the required information size (RIS). In other gene comparison models that yielded positive results, the cumulative Z curve did not cross the sequential trial monitoring boundary, and the sample size did not reach RIS, demonstrating that the pooled results possibly were not solid enough. Figure 5 shows the results of trial sequential analysis under the dominant model and the heterozygote model of rs2010963.

## Discussion

Polycystic ovary syndrome (PCOS) is a female endocrine disorder with complex etiology. Currently, the widely adopted diagnostic criteria are Rotterdam criteria: rare ovulation or anovulation, clinical or biochemical hyperandrogenemia, and ovarian polycystic changes determined by ultrasound. Patients with two of three in the criteria and without other diseases that may cause hyperandrogenemia could be diagnosed with PCOS [30]. The prominent pathophysiological features of PCOS are hyperplasia and hypervascularity of the ovarian theca interna and stroma [2]. Angiogenesis in the ovary is essential for follicular growth, ovulation, and subsequent luteal development and regression. Increasing evidence demonstrates that there are multiple dysregulation of angiogenic factors in PCOS, including vascular endothelial growth factor, angiogenin, platelet-derived growth factor, transforming growth factor-alpha, and basic fibroblast growth factor. VEGF is one of the major regulators of angiogenesis, and its important role in physiological and pathological angiogenesis has been proved. It is the most extensively studied angiogenic factor related to the pathophysiology of PCOS [35]. In the female reproductive system, macrophages and granulosa cells are the main sources of VEGF production. As a homologous dimeric glycoprotein hormone that binds heparin, VEGF can effectively promote vascular formation and improve vascular permeability [36]. VEGF protein family consists of 6 members, namely VEGF (also known as VEGFA), VEGFB, VEGFC, VEGFD, VEGFE and placenta growth factor (PLGF), with molecular weight ranging from 35 kDa to 45 kDa. VEGF is recognized as a “central regulator” of angiogenesis due to its ability to specifically promote vascular endothelial division, proliferation and migration, and is the most potent angiogenic factor [36–39].

VEGF protein is known to be closely related to the pathogenesis of PCOS. Serum VEGF levels are significantly elevated in PCOS patients [16–18]. High levels of serum VEGF result in a higher number of active granuloas lutein cells (GLCs), increase secretion of GLCs, and up-regulate VEGF gene expression [40]. In addition, the expression of VEGF in the stromal hyperplasia of PCOS is significantly increased, resulting in an increase in microvascular permeability, and a large number of neovascularization may lead to dysplasia of the ovarian producing androgen. VEGF gene is located at 6p21.3, with a total length of about 14 KB, and is alternately composed of 8 exons and 7 introns. There are at least 30 single nucleotide polymorphisms (SNPs) in the coding region of VEGF gene. Based on previous studies, rs2010963 and rs3025039 mutation sites have been proved to alter the plasma level of VEGF [41, 42]. The correlation between VEGF gene polymorphisms and the risk of PCOS has attracted growing attention in recent years, among which the most widely investigated SNPs are rs2010963 (G > C), rs833061 (T > C), rs3025039 (C > T), rs699947 (A > C), and rs1570360 (A > G), but the results from different studies remain highly controversial. For example, in studies on the correlation between rs2010963 and PCOS susceptibility, 3 studies reported no significant correlation between the two, while the other 3 studies showed a reduced risk of PCOS in C allele carriers. In the studies on the correlation between rs833061 and PCOS susceptibility, except that BAO found that there was a significant statistical significance between this SNP and PCOS susceptibility, all the other 5 studies revealed that it was not related to the risk of PCOS. Therefore, this meta-analysis was conducted to comprehensively evaluate the relationship between VEGF gene polymorphisms and PCOS risk based on the data in the existing literature. According to the NOS standard, all 10 incorporated studies were considered to have high-quality. In the present study, the results of the pooled analysis showed a significant correlation between VEGF rs2010963 variability and decreased susceptibility to PCOS. Subgroup analysis based on different ethnic stratification showed that the negative correlation between rs2010963 mutation and PCOS susceptibility only appeared in the Asian races, and there was no significant correlation in the Caucasian populations. The pooled analysis of the association between the VEGF gene rs3025039 polymorphism and the risk of PCOS showed that this SNP increased the risk of PCOS in the general population. Subgroup analysis based on different ethnicities showed that the rs3025039 polymorphism in the Caucasian populations was also associated with an increased risk of PCOS. In the Asian populations, the VEGF gene rs3025039 polymorphism reduced the risk of PCOS. Therefore, our results showed that the correlation between VEGF gene polymorphisms and the risk of PCOS in different populations may be quite different, suggesting that further experimental studies are needed to explore the possible underlying reasons for this difference.

It must be recognized that this meta-analysis has some possible limitations. First, the number of articles included in the study was small, and the overall number of patients in the case group and control group was low. Some subgroup analyses were unable to perform data analysis because only one study was included, thus limiting the reliability of summary results. Secondly, the previous studies on VEGF gene polymorphisms and PCOS risk only contained two ethnicities, lacking relevant studies on other races. Third, although the inclusion criteria for the control group were clearly stated in all studies, the inclusion criteria for different studies were not the same. For example, in some studies, the inclusion criteria for the control group were healthy women of childbearing age, while in others the criteria detailed a history of successful childbearing and a recent absence of any medications that might affect hormone levels. As PCOS is a disease with complex etiology, its incidence is related to many factors, such as obesity and insulin resistance. Due to the absence of relevant patient data based on the two factors in the original study literature, we did not conduct subgroup stratification analysis based on factors such as whether the patient was obese or not and the severity of insulin resistance.

Despite some limitations, this meta-analysis still has several highlights. To date, this is the first known meta-analysis focused on the correlation between VEGF gene polymorphisms and PCOS risk. Through the establishment of strict inclusion and exclusion criteria, a total of 10 studies were included in this meta-analysis, involving 1347 PCOS cases and 1378 controls. The study spanned seven regions and included both Caucasian and Asian populations. We systematically evaluated the effect of VEGF gene polymorphism on the occurrence of PCOS. At the same time, in most gene comparison models, the calculated inter-study heterogeneity was low, indicating the results of this meta-analysis were stable. In addition, the results of trial sequential analysis (TSA) also demonstrate the correlation between rs2010963 and decreased risk of PCOS obtained through this current meta-analysis was stable and reliable. Many previous studies have suggested that multiple SNPs of VEGF gene increase the risk of diseases such as ovarian malignancies, hypertensive disorder of pregnancy, and idiopathic recurrent pregnancy loss, and thereafter have been confirmed by multiple meta-analyses. Our meta-analysis results revealed certain VEGF gene polymorphisms had a distinct different impact on the pathogenesis of PCOS in contrast to its role in the pathogenesis of other female reproductive system disorders [43–45]. There was a significant correlation between VEGF rs2010963 and decreased PCOS susceptibility, suggesting VEGF rs2010963 could be a protective factor for the risk of PCOS.

In conclusion, this study establishes a significant correlation between the variation of VEGF rs2010963 and decreased PCOS susceptibility in the entire population; subgroup analysis shows this association only occurs occurred in Asian populations. The mutation of VEGF gene rs3025039 may also decrease the risk of PCOS in the overall population; subgroup analysis based on different populations shows that rs3025039 polymorphism is also associated with decreased risk of PCOS in the Asian populations, while in the Caucasian populations rs3025039 polymorphism of VEGF gene enhances the risk of PCOS. More well-designed studies involving more cases will be needed in the future to confirm this inference.

## Data Availability

The current study was based on results of relevant published studies.

## Abbreviations

CI: Confidence interval
HWE: Hardy–Weinberg equilibrium
OR: Odds ratio
PCOS: Polycystic ovarian syndrome
SNP: Single nucleotide polymorphism
VEGF: Vascular endothelial growth factor
